# Partial splenic embolization as a rescue and emergency treatment for portal hypertension and gastroesophageal variceal hemorrhage

**DOI:** 10.1186/s12876-023-02808-1

**Published:** 2023-05-24

**Authors:** Vlad Pavel, Gregor Scharf, Patricia Mester, Lea U. Krauss, Karsten Gülow, Alexander Mehrl, Martina Müller, Stephan Schmid

**Affiliations:** 1 Department of Internal Medicine I, Gastroenterology, Hepatology, Endocrinology, Rheumatology and Infectious Diseases, University Hospital Regensburg, Franz-Josef-Strauß-Allee 11, 93053 Regensburg, Germany; 2 Department of Radiology, University Hospital Regensburg, Regensburg, Germany

**Keywords:** Partial splenic embolization, Portal hypertension, Esophageal varices, Gastric varices, Gastrointestinal hemorrhage, Liver cirrhosis, Acute-on-chronic liver failure, Cirrhotic portal hypertension, Non-cirrhotic portal hypertension

## Abstract

**Background:**

Partial splenic embolization (PSE) is a non-surgical procedure which was initially used to treat hypersplenism. Furthermore, partial splenic embolization can be used for the treatment of different conditions, including gastroesophageal variceal hemorrhage. Here, we evaluated the safety and efficacy of emergency and non-emergency PSE in patients with gastroesophageal variceal hemorrhage and recurrent portal hypertensive gastropathy bleeding due to cirrhotic (CPH) and non-cirrhotic portal hypertension (NCPH).

**Methods:**

From December 2014 to July 2022, twenty-five patients with persistent esophageal variceal hemorrhage (EVH) and gastric variceal hemorrhage (GVH), recurrent EVH and GVH, controlled EVH with a high risk of recurrent bleeding, controlled GVH with a high risk of rebleeding, and portal hypertensive gastropathy due to CPH and NCPH underwent emergency and non-emergency PSE. PSE for treatment of persistent EVH and GVH was defined as emergency PSE. In all patients pharmacological and endoscopic treatment alone had not been sufficient to control variceal bleeding, and the placement of a transjugular intrahepatic portosystemic shunt (TIPS) was contraindicated, not reasonable due to portal hemodynamics, or TIPS failure with recurrent esophageal bleeding had occurred. The patients were followed-up for six months.

**Results:**

All twenty-five patients, 12 with CPH and 13 with NCPH were successfully treated with PSE. In 13 out of 25 (52%) patients, PSE was performed under emergency conditions due to persistent EVH and GVH, clearly stopping the bleeding. Follow-up gastroscopy showed a significant regression of esophageal and gastric varices, classified as grade II or lower according to Paquet’s classification after PSE in comparison to grade III to IV before PSE. During the follow-up period, no variceal re-bleeding occurred, neither in patients who were treated under emergency conditions nor in patients with non-emergency PSE. Furthermore, platelet count increased starting from day one after PSE, and after one week, thrombocyte levels had improved significantly. After six months, there was a sustained increase in the thrombocyte count at significantly higher levels. Fever, abdominal pain, and an increase in leucocyte count were transient side effects of the procedure. Severe complications were not observed.

**Conclusion:**

This is the first study analyzing the efficacy of emergency and non-emergency PSE for the treatment of gastroesophageal hemorrhage and recurrent portal hypertensive gastropathy bleeding in patients with CPH and NCPH. We show that PSE is a successful rescue therapy for patients in whom pharmacological and endoscopic treatment options fail and the placement of a TIPS is contraindicated. In critically ill CPH and NCPH patients with fulminant gastroesophageal variceal bleeding, PSE showed good results and is therefore an effective tool for the rescue and emergency management of gastroesophageal hemorrhage.

**Supplementary Information:**

The online version contains supplementary material available at 10.1186/s12876-023-02808-1.

## Background

Gastroesophageal variceal hemorrhage is the most common and sometimes fatal complication of portal hypertension (PH) [1]. Portal hypertension can be classified as cirrhotic or non-cirrhotic (CPH and NCPH). Non-cirrhotic portal hypertension (NCPH) is the second leading cause of portal hypertension [2, 3]. The etiology of NCPH comprises the following five categories: 1. chronic infections, 2. exposure to medication, 3. thrombophilia, 4. immunological disorders, and 5. genetic disorders. Chronic abdominal infection is the most important etiological factor in eastern patients and thrombophilia in western patients. NCPH is characterized by features of portal hypertension, and moderate to massive splenomegaly, with or without hypersplenism. Of note, liver function is preserved in NCPH [3, 4]. The majority of patients with NCPH present with complications of PH, mainly splenomegaly and variceal bleeding.

Gastroesophageal varices are present in half of the patients with CPH. Between 25 to 35% of the patients with liver cirrhosis suffer from gastroesophageal hemorrhage [5]. Complications of PH, such as variceal hemorrhage, are of exceptionally high relevance for patients with liver cirrhosis, as they often constitute a progression from compensated cirrhosis to acute decompensation [6]. In more than 50% of CPH patients with upper gastrointestinal bleeding, concurrent infections occur within two weeks, which can lead to the development of acute-on-chronic liver failure (ACLF), a syndrome characterized by intra- and extrahepatic organ failure and a high short-term mortality [7].

Acute variceal bleeding is treated using pharmacological, endoscopic, and angiographic techniques as soon as the patient gains hemodynamic stability and airway protection [8]. Endoscopic band ligation is the treatment of choice in case of esophageal variceal bleeding and represents the most effective procedure [9]. In patients with acute hemorrhage of gastric varices, injection of tissue adhesive (cyanoacrylate) or endoscopic band ligation can be performed [10]. In severe cases with refractory gastroesophageal variceal hemorrhage, endoscopic placement of a self-expanding, fully silicone-covered nitinol stent system or a temporary balloon tamponade can be performed. Data show that the placement of a self-expanding covered esophageal metal stents is more efficacious and a safer option than balloon tamponade [9].

Furthermore, in patients with persistent esophageal variceal hemorrhage (EVH) and persistent gastric variceal hemorrhage (GVH), recurrent EVH and GVH, and controlled EVH and GVH with a high risk of rebleeding, after an initial pharmacological and endoscopic therapy, angiographic procedures like TIPS or balloon-occluded retrograde transvenous obliteration (BRTO) should be considered [9, 10]. In accordance with the European Association for the Study of the Liver (EASL) Clinical Practice Guidelines for the management of patients with decompensated cirrhosis, careful selection of patients for TIPS insertion is essential. The guideline specifies that TIPS is not recommended in patients with serum bilirubin > 5 mg/dl and current hepatic encephalopathy grade ≥ 2 or chronic hepatic encephalopathy, concomitant active infection, severe systolic or diastolic dysfunction, or pulmonary hypertension [11]. Moreover, TIPS insertion can be technically impossible or not reasonable due to portal hemodynamics.

An alternative endovascular strategy to reduce portal hypertension in patients with acute or recurrent variceal bleeding is the partial embolization of the splenic artery (PSE). In the latest guideline on endoscopic diagnosis and management of esophagogastric variceal hemorrhage of the European Society of Gastrointestinal Endoscopy (ESGE), PSE is not included in the respective treatment algorithms [10]. Splenic artery embolization was first described in 1973 by Frank E. Maddison. He performed a total splenic artery embolization using an autologous clot to treat hypersplenism [12–14]. Despite Maddison’s early success, severe complications of total splenic artery embolization have been reported. Complications like a splenic abscess, overwhelming pneumonia, hematoma, pancreatic infarction, or sepsis occurred [15]. In 1979 a PSE was successfully performed in six patients with variceal bleeding by Spigos et al. Severe complications were not observed. Since then, interventionalists have practiced a partial splenic embolization to preserve part of the splenic tissue for immunologic function [15–17]. These techniques became more widely used in the late 1990s [18]. A meta-analysis showed that the application of PSE, particularly when it is combined with endoscopic treatment, may play an important role in the management of esophagogastric variceal hemorrhage in the future [5]. This is supported by an article by Ishikawa et al., who conclude that from the perspectives of portal hemodynamics and hepatic function, PSE before or after endoscopic treatment could prevent posttreatment variceal recurrence [19].

Further indications of PSE are control of bleeding in blunt splenic injuries and hypersplenism due to various etiologies. PSE is nowadays a safe and effective alternative to splenectomy in many cases. Especially in patients with severe comorbidities like splenomegaly and liver cirrhosis, anemia, leukopenia, and thrombocytopenia improved after PSE [15, 17].

None of the studies analyzed the efficacy of PSE in an emergency situation of acute gastroesophageal hemorrhage. Therefore, we analyzed whether PSE presents a successful rescue therapy for patients with emergency and non-emergency gastroesophageal variceal hemorrhage due cirrhotic and non-cirrhotic PH in whom pharmacological or endoscopic treatment options failed or placement of a TIPS was contraindicated or technically not feasible.

## Material and Methods

### Aim of the study

This study aimed to evaluate the outcome and safety of partial splenic embolization (PSE) as a rescue treatment for portal hypertension (PH) and variceal hemorrhage in emergency and non-emergency patients with cirrhotic and non-cirrhotic PH. PSE was performed, when nonspecific beta-blocker (NSBB)-, and endoscopic treatment had failed, and the placement of a transjugular intrahepatic portosystemic shunt (TIPS) was contraindicated according to the Clinical Practice Guidelines of the European Association for the Study of the Liver (EASL) [11, 20].

### Study design and patient characteristics

This is a unicentric retrospective study. Patient data were collected from medical files, including ICU reports and discharge letters, as well as radiology and endoscopic reports. The information was gathered from the University Hospital Regensburg’s institutional archive and database (SAP® Version 7.50, SAP® SE, Walldorf, Germany, and Metavision®, iMDsoft®, Düsseldorf, Germany). Furthermore, demographical data such as age, gender, and body weight, as well as medical history, were recorded.

**Fig. 1:**
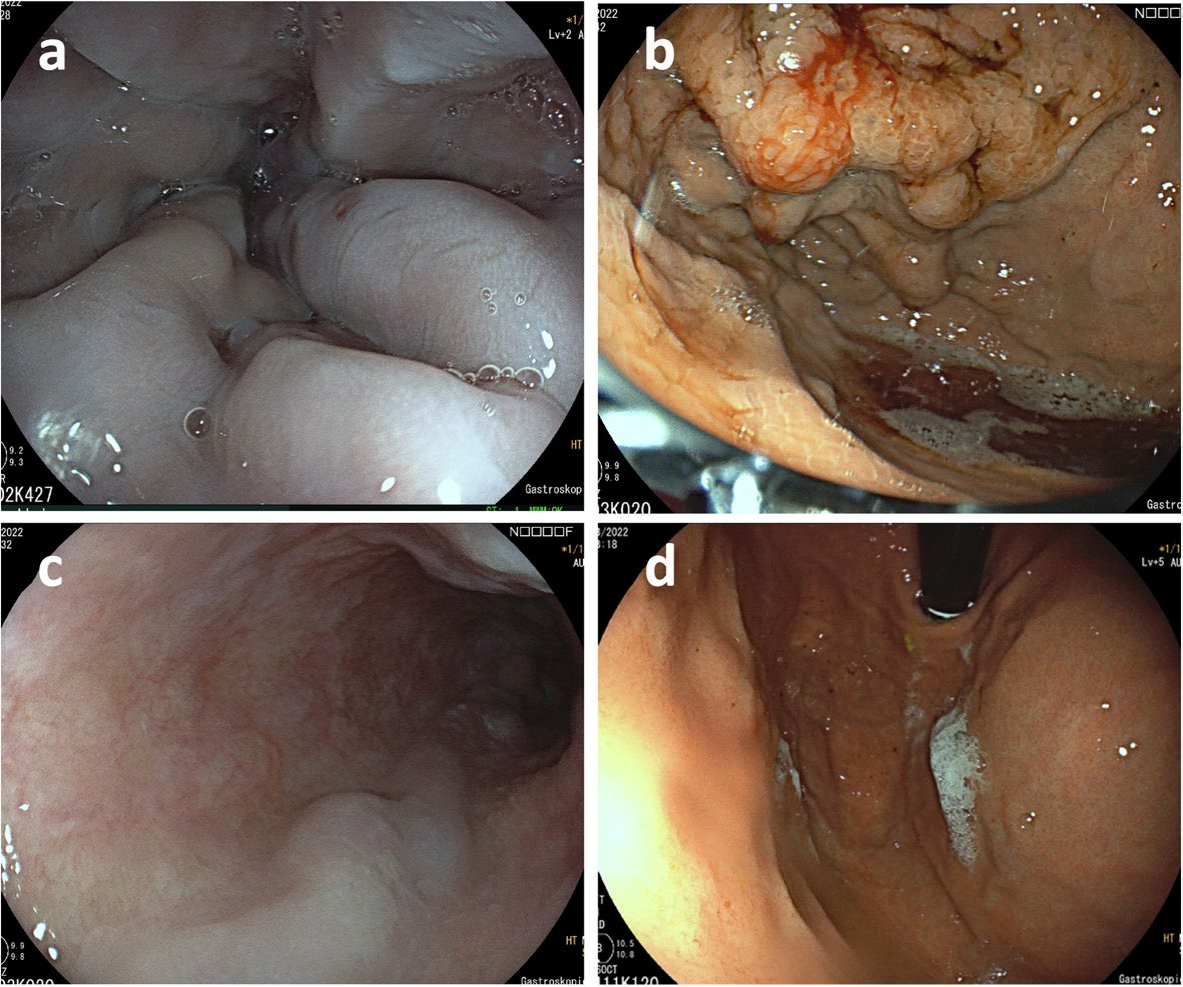
Esophageal varices grade III (**a**) and fundal varices (**b**) before partial splenic artery embolization. Regression of esophageal varices to grade I-II (**c**) and disappearance of fundal varices (**d**) after partial splenic artery embolization

From December 2014 to July 2022, twenty-five patients with cirrhotic and non-cirrhotic PH and persistent esophageal variceal hemorrhage (EVH) and gastric variceal hemorrhage (GVH), recurrent EVH and GVH, controlled EVH with a high risk of recurrent bleeding, controlled GVH with a high risk of rebleeding and portal hypertensive gastropathy bleeding underwent emergency and non-emergency PSE (Fig. 1). PSE for treatment of persistent EVH and GVH was defined as emergency PSE according to the current European Society of Gastrointestinal Endoscopy (ESGE) Guideline, and cirrhotic (CPH) and non-cirrhotic portal hypertension (NCPH) were defined according to literature, subgroup analyses were performed [10, 21–26].

During the hospitalization of the patients, the clinical management was performed following the respective guidelines of the national and international expert societies [9–11, 27].

Esophageal varices were classified according to Paquet. Esophageal varices grade I are microcapillaries in the distal esophagus or at the esophagogastric junction. Esophageal varices grade II are one or two small varices in the distal esophagus. Grade III esophageal varices are described as medium-sized varices of any number, and grade IV varices as large-sized varices in any part of the esophagus [28, 29]. All patients included in the study had esophageal varices grade III and IV before PSE was performed.

Twelve patients in the study group had underlying liver cirrhosis. To characterize the disease severity of these patients with liver cirrhosis, the Chronic Liver Failure Consortium (CLIF)-C ACLF score, a score derived and validated by the CLIF consortium, and the Model for End-stage Liver Disease (MELD) score were used [30–32].

The following formula was used for the calculation of the CLIF-C ACLF Score, wherein the CLIF-C OF score was raised according to [32]: CLIF-C ACLF score = 10 × (0,33 × CLIF-C-OFs + 0,04 × Age + 0,63 × ln (WBC count in 10^3^/μl) –2).

The MELD score was calculated for each patient using the following Eq. [30, 31]: MELD score = 9.57 × ln (serum creatinine) + 3.78 ln (total bilirubin) + 11.2 × ln (international normalized ratio) + 6.43.

### The procedure of partial splenic embolization (PSE)

Informed consent was obtained from all patients or family members. In thirteen cases (52%), the procedure was done under emergency conditions due to severe and otherwise uncontrollable gastroesophageal variceal hemorrhage. On average, a 60–80% spleen embolization was achieved by PSE.

**Fig. 2:**
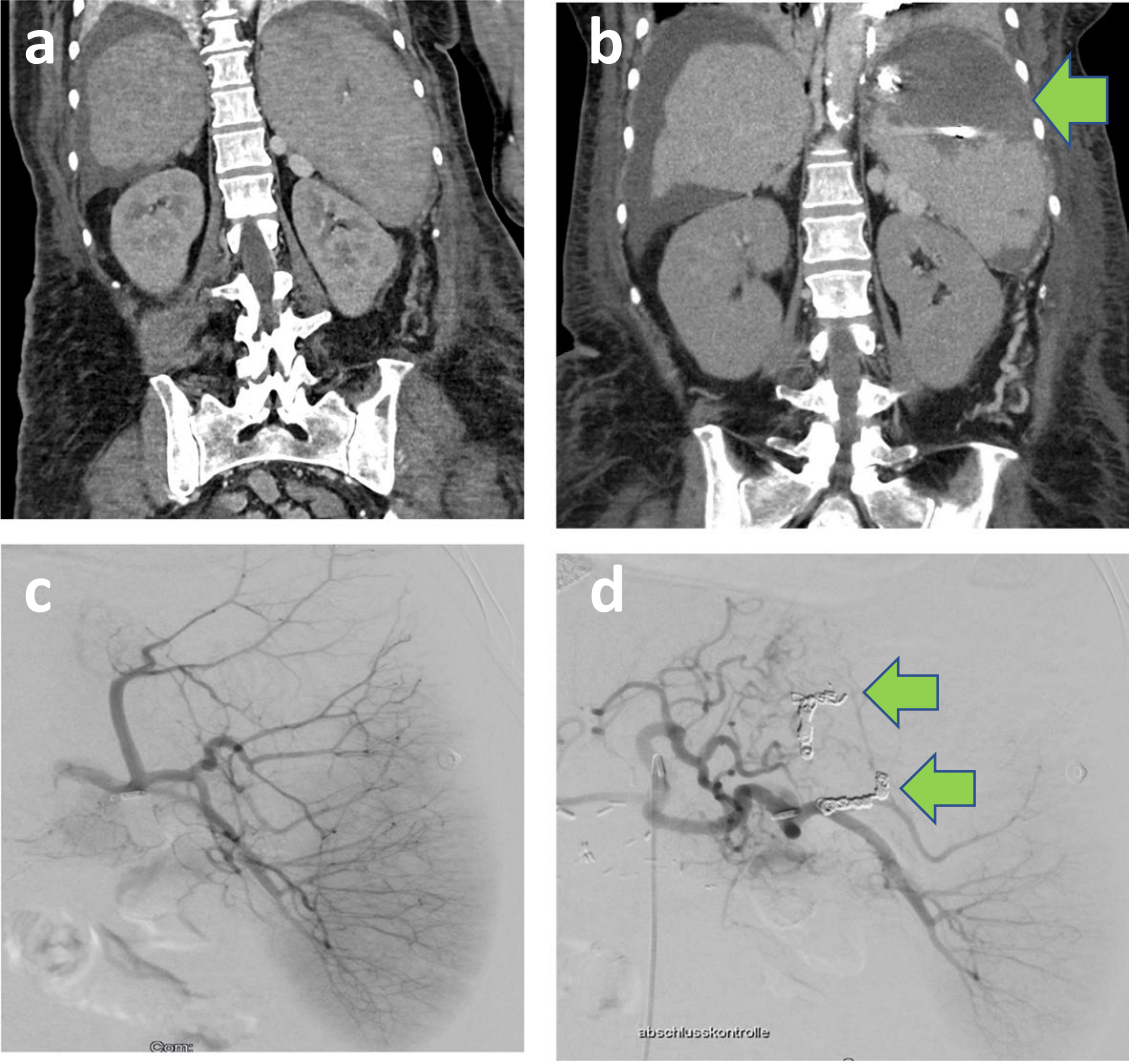
Computed tomography of a patient before (**a**) and after (**b**) partial splenic artery embolization. Angiography before (**c**) and after (**d**) partial splenic artery embolization. Embolization of the upper branches of the splenic arteries using microcoils and gelatin (marked with arrows); embolization of 60% of the splenic parenchyma d (marked with arrow)

Under a strict aseptic technique, a percutaneous femoral artery approach was used. The splenic artery was catheterized using a 4 French or 5 French Cobra Catheter (Cordis, Miami Lakes, FL, USA). A 2.7 French Progreat® Microcatheter (Terumo Corporation, Tokyo, Japan) was introduced through the Cobra® catheter into the intrasplenic arterial branches. The upper intrasplenic branches were chosen for embolization (Fig. 2). The branches were embolized by different-sized platinum micro coils according to vessel size. If persistent filling of the treated branches was observed, a mixture of EmboCube® Embolization Gelatin (Merit Medical Systems, South Jordan, UT, USA) and contrast media was additionally used for the embolization. Final angiography showed good results post-interventionally, with 60–80% splenic embolization as a result of the intervention.

### Follow-up after partial splenic embolization

To re-evaluate and monitor the variceal status after the partial splenic artery embolization, control gastroscopies were performed. In addition, to evaluate the extent of the splenic necrosis after embolization and to rule out abscess formation, sonography was performed. The patients were followed for six months after PSE.

### Statistical analysis

Statistical analysis was performed using Microsoft Excel® (Microsoft, Redmond, WA, USA).and SigmaPlot® 14.5 (Systat Software, San Jose, CA, USA). To assess statistical analysis, the Mann–Whitney Rank Sum Test was used. A *p*-value under 0.05 was considered significant.

## Results

### Baseline characteristics of the patients who underwent PSE

**Fig. 3:**
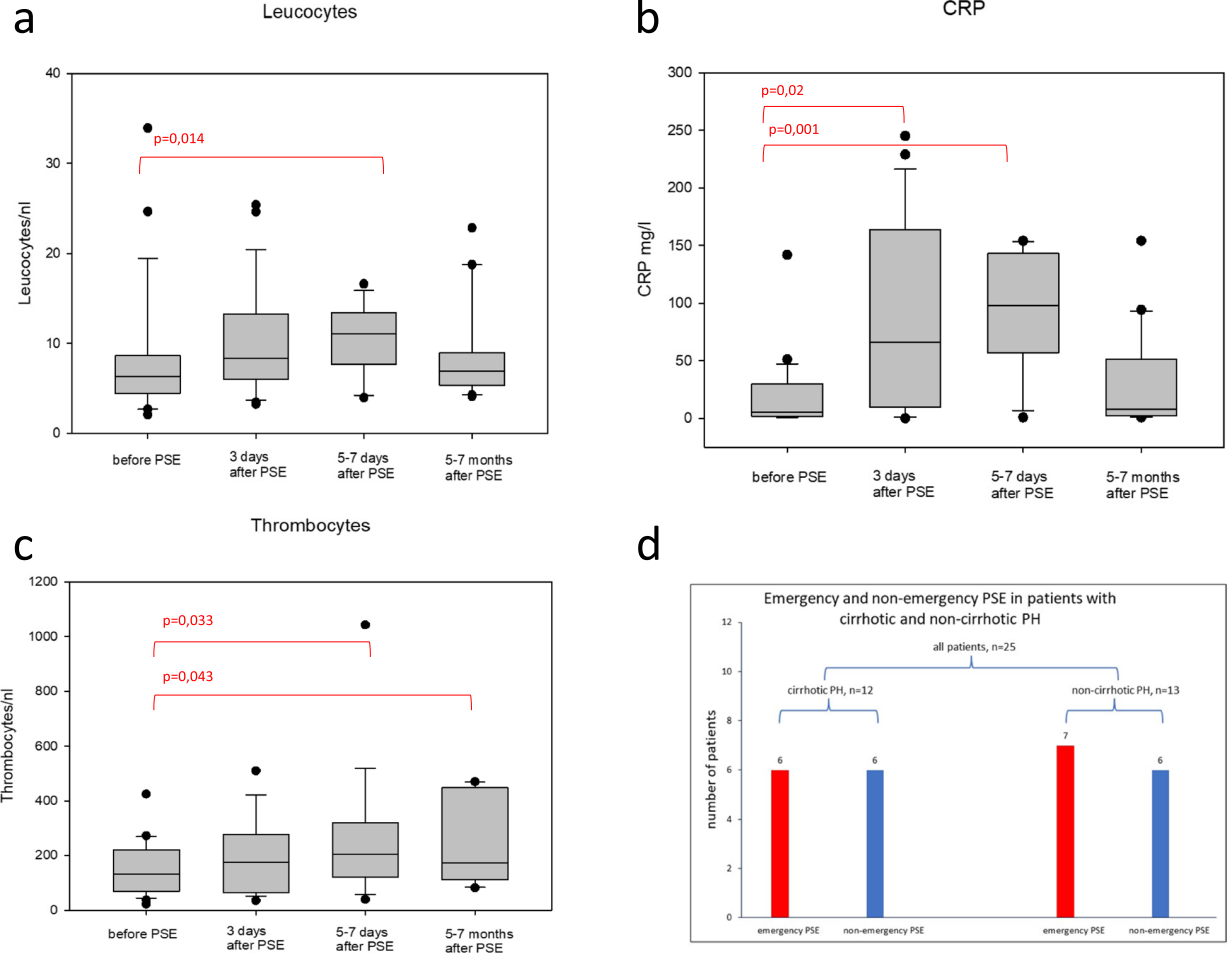
Leucocytes and CRP levels temporarily rising initially after partial splenic artery embolization, decreasing again after six months (**a **and** b**). Significant increase of thrombocyte levels after partial splenic artery embolization, slightly decreasing after 5–7 months, still remaining significantly higher compared to the levels before the intervention (**c**). Distinction of PSE performed in emergency and non-emergency situations in patients with cirrhotic (CPH) and non-cirrhotic portal hypertension (NCPH) (**d**)

PSE was successfully performed in all twenty-five patients. The median age of the patients was 57 years, six were female, and nineteen were male. Twelve patients had cirrhotic portal hypertension (CPH), and thirteen had non-cirrhotic portal hypertension (NCPH). Of the patients with CPH, four patients each had Child A, B, and C cirrhosis. The median bilirubin level was 2.75 mg/dl, and a median MELD Score of 19.0 was calculated. Analyses of the CLIF Consortium acute-on-chronic liver failure (CLIF-C ACLF) score showed a median of 42.5. Of the patients with CPH, six presented with emergency variceal hemorrhage. Seven of the patients with NCPH had emergency variceal hemorrhage (Fig. 3d). The etiology of NCPH was due to myeloproliferative neoplasia in 6 patients, idiopathic portal vein thrombosis in four patients, and pancreatic cancer, chronic myeloid leukemia, and chronic pancreatitis in one patient each.

No severe complications of PSE like splenic rupture, abscess, pneumonia, or PSE-related sepsis were observed. This also applies to patients who were treated under emergency conditions. Nineteen men and six women underwent PSE; the patients were between 18 and 77 years old. The patient's age, gender, etiology of portal hypertension, and indications for PSE are summarized in Table 1.

**Table 1:** Clinical characteristics of the study cohort

Characteristics	total study population (*n* = 25)
**Age** [years]: median (range)	57 (18–78)
**Sex**:	
Female n (%)	6 (24)
Male n (%)	19 (76)
**Etiology of portal hypertension:**	
**Cirrhotic portal hypertension n (%)**	**12 (48.0)**
Emergency variceal hemorrhage n (%)	6 (50.0)
Child–Pugh Score A n, (median, range)	4 (5.5, 5.0–6.0)
Child–Pugh Score B n, (median, range)	4 (7.5, 7.0–9.0)
Child–Pugh Score C n, (median, range)	4 (14.0, 12.0–15.0)
Bilirubin level: median (range), mg/dl	2.75 (0.2–10.2)
MELD Score: median (range)	19.0 (8.0–32.0)
CLIF-C ACLF score: median (range)	42.5 (20.0–71.0)
**Non-cirrhotic portal hypertension n (%)**	**13 (52.0)**
Emergency variceal hemorrhage n (%)	7 (53.9)
Myeloproliferative neoplasia n (%)	6 (46.1)
Idiopathic portal vein thrombosis n (%)	4 (30.8)
Pancreatic cancer n (%)	1 (7.7)
Chronic myeloid leukemia n (%)	1 (7.7)
Chronic pancreatitis n (%)	1 (7.7)

Presentation of the baseline demographic (age, sex) and clinical characteristics, including etiology of portal hypertension of the study cohort (*n* = 25), as well as the Child–Pugh Score, Bilirubin levels, MELD Score, CLIF Consortium acute-on-chronic liver failure (CLIF-C ACLF) score of the subgroup of patients with cirrhotic portal hypertension (*n* = 12)

Due to persistent EVH and GVH (13 patients), recurrent EVH (2 patients), controlled EVH with high risk of recurrent bleeding (8 patients), controlled GVH with high risk of rebleeding (1 patient), and recurrent portal hypertensive gastropathy bleeding (1 patient), pharmacological and endoscopic treatment were not sufficient to adequately control the GI bleeding. Upon presentation with variceal bleeding, all patients with previously known esophageal or gastric varices (21 patients) and no contraindication received long-term treatment with non-specific beta-blockers (NSBBs). Carvedilol was administered in fourteen patients, and Propranolol in seven patients. Four patients had no long-term NSBB therapy as esophageal or gastric varices had not been previously diagnosed (3 patients) or long-term NSBB therapy was not tolerated due to arterial hypotension (1 patient). In all included patients—CPH and NCPH patients -, placement of a transjugular intrahepatic portosystemic shunt (TIPS) was contraindicated. As shown in Table 2 TIPS was anatomically unfeasible in thirteen patients, Bilirubin was higher than 5 mg/dl in three patients, TIPS was not appropriate due to portal hemodynamics in six patients, TIPS failure with recurrent esophageal bleeding occurred in two patients, and right heart failure was a contraindication for TIPS in one patient.

**Table 2:** Indications for partial splenic embolization (PSE)

	**cirrhotic PH, *n* = 12**	**non-cirrhotic PH, *n* = 13**	**all patients, *n* = 25**
**PSE indications:** n (%)			
Persistent EVH	4 (33.3%)	3 (23.1%)	7 (28.0%)
Persistent GVH	2 (16.7%)	4 (30.8%)	6 (24.0%)
Recurrent EVH	1 (8.3%)	1 (7,7%)	2 (8%)
Recurrent GVH	0 (0.0%)	0 (0.0%)	0 (0.0%)
Controlled EVH with high risk of recurrent bleeding	4 (33.3%)	4 (30.8%)	8 (32.0%)
Controlled GVH with high risk of rebleeding	0 (0.0%)	1 (7.7%)	1 (4.0%)
Recurrent portal hypertensive gastropathy bleeding	1 (8.3%)	0 (0.0%)	1 (4.0%)
**NSBB failure:** n (%)	10 (83.3%)	11 (84.6%)	21 (84.0%)
**TIPS contraindications:** n (%)			
TIPS anatomically not possible	4 (33.3)	9 (69.2)	13 (52.0)
Bilirubin > 5 mg/dl	3 (25.0)	0 (0.0)	3 (12.0)
TIPS not reasonable due to portal hemodynamics	2 (16.7)	4 (30.8)	6 (24.0)
TIPS failure with recurrent variceal bleeding	2 (16.7)	0 (0.0)	2 (8.0)
Right heart failure	1 (8.4)	0 (0.0)	1 (4.0)

Presentation of indications for partial splenic embolization (PSE), failure of nonspecific beta-blockers (NSBB), and contraindications for transjugular intrahepatic portosystemic shunt (TIPS) for cirrhotic and non-cirrhotic portal hypertension (PH). Classification of esophageal variceal hemorrhage (EVH) and gastric variceal hemorrhage (GVH) according to the ESGE Guideline on endoscopic diagnosis and management of esophagogastric variceal hemorrhage (2022). Persistent EVH and GVH were defined as emergency variceal hemorrhage. Contraindications for TIPS are presented according to the EASL Clinical Practice Guidelines for the management of patients with decompensated cirrhosis (2018) and EASL Clinical Practice Guidelines on prevention and management of bleeding and thrombosis in patients with cirrhosis (2022)

### PSE as an emergency procedure in cirrhotic (CPH) and non-cirrhotic portal hypertensive (NCPH) hemorrhage

In our cohort, PSE was predominantly (13 out of 25 patients) performed as an emergency procedure in CPH (6 patients) and NCPH (7 patients). Of the thirteen patients treated with emergency PSE, seven had persistent EVH, and six had persistent GVH. As in the total cohort, TIPS was contraindicated in these critically ill patients. PSE under emergency conditions controlled persistent EVH and GVH without severe complications in all patients with acute gastroesophageal hemorrhage caused by cirrhotic and non-cirrhotic portal hypertension.

### Laboratory findings after PSE

Due to different underlying conditions, like liver cirrhosis, in twelve patients, the laboratory findings showed thrombocytopenia. Platelet count increased on day one after PSE, and after one week, thrombocyte levels improved significantly from 22–425/nl (median 142.5/nl) before PSE up to 105–1043/nl (median 247/nl) after the procedure. The increase in platelet count persisted over time and was confirmed after 5–7 months (Fig. 3).

### Analysis of incidence and treatment of postembolization syndrome (PES)

All patients were hospitalized after the procedure, and potential adverse effects were recorded and treated. Patients were especially monitored for the development of postembolization syndrome (PES). Conservative management included antibiotics, appropriate hydro electrolytic infusion, and analgesic treatment. Peripheral blood count and inflammatory markers were controlled daily after the procedure.

Twelve of 25 patients have experienced PES in different degrees after PSE (Table 3). Six patients (24.0%) were febrile, and another three patients (12.0%) complained about abdominal pain, and pain medications were necessary to relieve the symptoms. Left-sided pleural effusion occurred in two patients (8.0%) after the procedure. To drain the pleural effusion, one patient needed pleural drainage. The other patient with pleural effusion was treated with diuretics only. Post-interventional ascites did not occur in our patients. One patient (4.0%) developed a small perisplenic hematoma which spontaneously regressed. No blood transfusion was mandatory during or related to PSE.

**Table 3:** Postembolization syndrome (PES) after partial splenic embolization (PSE)

Symptoms of PES	cirrhotic PH, *n* = 12	non-cirrhotic PH, *N* = 13	all patients, *n* = 25
Fever n (%)	2 (16.67)	4 (30.77)	6 (24.0)
Abdominal pain n (%)	1 (8.33)	2 (15.38)	3 (12.0)
Pleural effusion n (%)	1 (8.33)	1 (7.69)	2 (8.0)
Small hematoma n (%)	1 (8.33)	0 (0)	1 (4.0)
**No symptoms of PSE n (%)**	**7 (58.33)**	**6 (46.15)**	**13 (52.0)**

Incidence of the symptoms of PES (fever, abdominal pain, pleural effusion, small hematoma) in patients with cirrhotic portal hypertension (PH) (*n* = 12), non-cirrhotic PH (*n* = 13), and all patients (*n* = 25)

All patients developed elevated inflammatory parameters, but under antibiotic therapy, depending on the clinical condition, either with Piperacillin/Tazobactam, Meropenem, Linezolid, or Vancomycin, the inflammatory parameters were regressive. At the latest, after six months, inflammatory markers like C-reactive protein and leucocytes normalized (Fig. 3). No other significant pathological laboratory findings were observed after PSE.

### Outcome of PSE

No patient suffered from recurrent gastroesophageal variceal bleeding after PSE. Critically ill patients who needed emergency PSE due to gastroesophageal variceal hemorrhage stayed in the intensive care unit until hemodynamic stability was achieved. None of the patients developed severe PSE-related complications. A control gastroscopy showed a significantly better variceal status, now described as grade II or lower (Fig. 1), in comparison to the status of the gastroesophageal varices before the intervention (grade III to IV). Fundal varices were no longer detectable in 95.8% of the patients. Five patients needed a repeated PSE due to recurrent varices without rebleeding. Here, embolization of further superior or inferior intrasplenic branches, in addition to the initial embolization of the upper intrasplenic branches of the splenic artery, was performed.

Three patients died during the follow-up, but the death was unrelated to PSE. One patient with liver cirrhosis Child–Pugh C due to alcohol misuse, died four weeks after PSE due to ACLF caused by pneumonia with acute respiratory distress syndrome (ARDS). Complications of PSE were excluded using a CT scan and repeated sonographic examinations. One patient with CPH died due to end-stage hepatocellular carcinoma, another patient with NCPH died six months after the procedure due to ventricular fibrillation.

We show that PSE for rescue treatment of gastroesophageal variceal hemorrhage and recurrent portal hypertensive gastropathy bleeding is a safe procedure even in an emergency situation in patients with CPH and NCPH. No gastroesophageal variceal hemorrhage recurred after PSE, variceal status improved, and thrombocyte levels increased significantly.

## Discussion

Patients with portal hypertension often develop esophageal varices and gastric varices [33, 34]. In patients with gastroesophageal varices, pharmacologic and endoscopic treatment is used as first-line therapy. In patients with persistent esophageal variceal hemorrhage (EVH) and persistent gastric variceal hemorrhage (GVH), recurrent EVH and GVH, controlled EVH with a high risk of recurrent bleeding, and controlled GVH with a high risk of rebleeding, TIPS represents an effective treatment [10, 35]. However, sometimes due to severe portal vein thrombosis, cavernous transformation, and anatomical complexity, placement of a TIPS is not possible or has a high risk of complications. Furthermore, in patients with acute-on-chronic liver failure with hyperbilirubinemia or hepatic encephalopathy, placement of a TIPS is not the treatment of the first choice [36]. In these cases, when endoscopy is no longer promising or placement of a TIPS is not possible, PSE could be a good alternative (Fig. 2).

Established indications of PSE include hypersplenism with portal hypertension, thalassemia, autoimmune hemolytic anemia, hereditary spherocytosis, splenic trauma, and idiopathic thrombocytopenic purpura or splenic hemangioma [36]. The efficacy of PSE observed in our study is in line with the results of previously published studies or case reports in the management of patients with recurrent varices or variceal bleeding [37–42]. Not only had the patients no more bleeding episodes, but in addition, the hematological indices improved. All patients in our study improved their platelet count after the procedure. Furthermore, platelet levels remained significantly higher than before PSE. Previous studies also demonstrated improved and sustained platelet levels after PSE [43–49].

Of clinical relevance, we show that even in patients with endoscopically uncontrollable gastroesophageal variceal hemorrhage (= persistent EVH and GVH), PSE could be safely performed under emergency conditions. Thirteen of twenty-five patients underwent emergency PSE due to fulminant variceal bleeding. This is the first study analyzing the outcome of PSE under emergency conditions for gastroesophageal variceal hemorrhage, both in patients with CPH and in patients with NCPH. Our study shows excellent results. So far, there were only three case reports describing PSE under emergency conditions for variceal bleeding. Iwamoto and Shigemoto performed PSE in one patient with acute esophageal variceal bleeding due to CPH. The bleeding clearly stopped after the procedure [38]. Chikamori et al. describe in another case report an emergency hybrid procedure that combines endoscopic treatment with PSE for bleeding esophagogastric varices in two patients (1 patient with CPH and 1 patient with NCPH) [50]. In a case report published in 2010, Saugel et al. report successful PSE in a patient with bleeding gastric varices and splenic vein thrombosis in NCPH [51].

Although post-embolization syndrome was developed in 52.0% of our patients, it could be resolved rapidly in all patients after conservative treatment. Only one patient needed transient drainage for pleural effusion. Even though major complications of PSE, such as rupture of the spleen, splenic abscess, pneumonia, and septicemia, have been reported by other authors [52, 53], we did not observe any severe complications in our patients. The study of Owman et al. showed a positive correlation between the risk of splenic abscess and the volume of spleen necrosis induced by PSE [54]. To avoid major complications, the authors recommended only a 50% reduction of the splenic volume. However, the study conducted by Hayashi et al. demonstrated that a 70–80% PSE is a safe procedure. Overall, no severe complications occurred in their study. Two patients developed pleural effusions (8.0%). Furthermore, the authors concluded that the increase in platelet count was essentially dependent on the infarcted splenic volume [28]. A recent study by Lu et al. shows, that the combined use of dexamethasone and low-molecular-weight heparin after PSE can significantly reduce the incidence of complications [55, 56]. Of note, our patients did not encounter any severe complications after 70–80% splenic embolization and showed a sustained decrease in their platelet count. All our patients received unfractionated heparin (UFH) after PSE, steroids were not applied.

In another study an 80% PSE was performed in patients with hypersplenism due to liver cirrhosis [57]. No patient developed pleural effusion. Their results indicated that pleural effusion might be preventable by the preservation of the splenic upper pole. Also, in their patient group, no septic complications developed in the long‑term follow‑up period.

Severe long-term complications like splenic vein thrombosis were not observed. Follow-up investigations did not show splenic vein thrombosis in any patient. All patients were free from symptoms during the follow-up period.

## Conclusion

Partial splenic embolization is a rescue option for CPH and NCPH patients in the emergency situation of persistent EVH and GVH and the non-emergency situation of recurrent EVH and GVH, controlled EVH with a high risk of recurrent bleeding, controlled GVH with a high risk of rebleeding where pharmacological and endoscopic treatment is not sufficient, and the placement of a TIPS is impossible. Furthermore, PSE improved thrombocytopenia in our study.

The indication for PSE should be evaluated interdisciplinary among hepatologists, endoscopists, and interventional radiologists. The extent of the splenic necrosis should be carefully controlled. Our study showed that 60–80% splenic embolization is a safe procedure. Septic complications were not observed. To achieve good results, a protocol of prophylactic antibiotics is suggested.

We recommend considering PSE as a rescue and emergency treatment in cases of gastroesophageal variceal hemorrhage when pharmacological therapy and endoscopy fail, and placement of a TIPS is not possible or contraindicated.

## Supplementary Information

**Additional file 1: Supplementary Table 1. **Detailed characterization ofthe patient cohort

## Data Availability

The datasets generated and/or analyzed during the current study are not publicly available due to data privacy but are available from the corresponding author on request.

## Abbreviations

ACLF: Acute-on-chronic liver failure
ARDS: Acute respiratory distress syndrome
BRTO: Balloon-occluded retrograde transvenous obliteration (BRTO)
CLIF-C: Chronic Liver Failure Consortium
CPH: Cirrhotic portal hypertension
CT: Computed tomography
EASL: European Association for the Study of the Liver
EVH: Esophageal variceal hemorrhage
GVH: Gastric variceal hemorrhage
NCPH: Non-cirrhotic portal hypertension
NSBB: Nonspecific beta-blocker
PES: Postembolization syndrome
PH: Portal hypertension
PSE: Partial splenic embolization
TIPS: Transjugular intrahepatic portosystemic shunt
UFH: Unfractionated heparin
