# Supplementary material for: Partial splenic embolization as a rescue and emergency treatment for portal hypertension and gastroesophageal variceal hemorrhage
Source: BMC Gastroenterol. 2023 May 24;23:180. doi: 10.1186/s12876-023-02808-1 (PMC10207732; doi:10.1186/s12876-023-02808-1)
Supplement: Supplementary file 1 — Additional file 1: Supplementary Table 1. Detailed characterization ofthe patient cohort [file 12876_2023_2808_MOESM1_ESM.docx]

**Supplementary Table: Detailed characterization of the patient cohort**

| **Patient** | **Sex** | **Age** | **Date of**  **PSE** | **Etiology of portal hypertension** | **Underlying Disease** | **Emergency/**  **Non-emergency** | **PSE**  **indication** | **Long-term use of NSBB** | **TIPS contraindication** | **PES** | **6 months survival** |
| --- | --- | --- | --- | --- | --- | --- | --- | --- | --- | --- | --- |
| 1 | M | 56 | 01.12.2014 | non-cirrhotic | MPN | Emergency | pGVH | Propranolol | TIPS anatomically not possible | fever | survived |
| 2 | F | 55 | 16.11.2016 | non-cirrhotic | MPN | Non-emergency | cEVH | Carvedilol | TIPS anatomically not possible | pleural effusion | survived |
| 3 | M | 23 | 24.11.2016 | cirrhotic | Liver cirrhosis Child A (5)  MELD 9  CLIF-C-ACLF 24 | Non-emergency | cEVH | Carvedilol | TIPS not reasonable due to portal hemodynamics | no PES | survived |
| 4 | M | 64 | 26.01.2017 | non-cirrhotic | MPN | Non-emergency | cEVH | Propranolol | TIPS anatomically not possible | no PES | survived |
| 5 | M | 59 | 06.07.2018 | cirrhotic | Liver cirrhosis Child A (5)  MELD 10  CLIF-C-ACLF 50 | Emergency | pGVH | No (varices previously not known) | Right heart failure | fever | survived |
| 6 | M | 48 | 30.07.2018 | non-cirrhotic | Pancreatic cancer | Emergency | pGVH | No (arterial hypotension) | TIPS not reasonable due to portal hemodynamics | no PES | survived |

| 7 | M | 74 | 28.08.2018 | non-cirrhotic | Chronic pancreatitis | Emergency | pGVH | Carvedilol | TIPS not reasonable due to portal hemodynamics | no PES | survived |
| --- | --- | --- | --- | --- | --- | --- | --- | --- | --- | --- | --- |
| 8 | M | 78 | 12.07.2019 | cirrhotic | Liver cirrhosis Child A (6)  MELD 8  CLIF-C-ACLF 43 | Emergency | pGVH | Propranolol | TIPS failure | abdominal pain | survived |
| 9 | M | 18 | 05.05.2020 | cirrhotic | Liver cirrhosis Child A (6)  MELD 22  CLIF-C-ACLF 20 | Non-emergency | cEVH | Propranolol | TIPS not reasonable due to portal hemodynamics | no PES | survived |
| 10 | F | 56 | 29.06.2020 | non-cirrhotic | MPN | Emergency | pEVH | Carvedilol | TIPS anatomically not possible | fever | survived |
| 11 | M | 34 | 25.11.2020 | non-cirrhotic | Idiopathic portal vein thrombosis | Emergency | pEVH | Carvedilol | TIPS anatomically not possible | fever | survived |
| 12 | M | 57 | 04.12.2020 | cirrhotic | Liver cirrhosis Child B (7)  MELD 11  CLIF-C-ACLF 34 | Emergency | pEVH | Carvedilol | TIPS anatomically not possible | no PES | survived |
| 13 | M | 50 | 12.05.2021 | non-cirrhotic | Chronic myeloid leukemia | Non-emergency | cGVH | Carvedilol | TIPS not reasonable due to portal hemodynamics | no PES | Died because of ventricular fibrillation |
| 14 | M | 50 | 09.06.2021 | non-cirrhotic | MPN | Emergency | pGVH | No (varices previously not known) | TIPS not reasonable due to portal hemodynamics | no PES | survived |
| 15 | M | 68 | 19.07.2021 | non-cirrhotic | MPN | Non-emergency | cEVH | Carvedilol | TIPS anatomically not possible | no PES | survived |
| 16 | F | 66 | 29.06.2021 | non-cirrhotic | MPN | Non-emergency | rEVH | Propranolol | TIPS anatomically not possible | fever | survived |
| 17 | M | 67 | 29.07.2021 | cirrhotic | Liver cirrhosis Child B (7)  MELD 15  CLIF-C-ACLF 39 | Non-emergency | rEVH | Propranolol | TIPS anatomically not possible | fever | survived |
| 18 | F | 62 | 22.02.2022 | cirrhotic | Liver cirrhosis Child C (12)  MELD 22  CLIF-C-ACLF 71 | Emergency | pEVH | Propranolol | Bilirubin > 5 mg/dl | no PES | Died because of ARDS and ACLF |
| 19 | M | 73 | 01.03.2022 | non-cirrhotic | Idiopathic portal vein thrombosis | Emergency | pEVH | Carvedilol | TIPS anatomically not possible | abdominal pain | survived |
| 20 | M | 53 | 04.03.2022 | non-cirrhotic | Idiopathic portal vein thrombosis | Non-emergency | cEVH | Carvedilol | TIPS anatomically not possible | abdominal pain | survived |
| 21 | M | 57 | 04.05.2022 | cirrhotic | Liver cirrhosis Child B (8)  MELD 19  CLIF-C-ACLF 39 | Non-emergency | cEVH | Carvedilol | TIPS anatomically not possible | no PES | survived |
| 22 | F | 53 | 16.05.2022 | cirrhotic | Liver cirrhosis Child C (14)  MELD 20  CLIF-C-ACLF 61 | Non-emergency | cEVH | No (varices previously not known) | Bilirubin > 5 mg/dl | small hematoma | survived |
| 23 | M | 66 | 17.05.2022 | cirrhotic | Liver cirrhosis Child C (15)  MELD 32  CLIF-C-ACLF 66 | Emergency | pEVH | Carvedilol | TIPS anatomically not possible | no PES | Died because of end stage HCC |
| 24 | M | 36 | 27.05.2022 | cirrhotic | Liver cirrhosis Child B (9)  MELD 25  CLIF-C-ACLF 42 | Emergency | pEVH | Carvedilol | Bilirubin > 5 mg/dl | pleural effusion | survived |
| 25 | F | 64 | 21.07.2022 | cirrhotic | Liver cirrhosis Child C (14)  MELD 19  CLIF-C-ACLF 47 | Non-emergency | PHG | Carvedilol | TIPS failure | no PES | survived |

Presentation of patient characteristics, including date of PSE, etiology of portal hypertension, underlying disease, emergency/non-emergency PSE, long-term use of NSBBs, TIPS contraindications, PES, and 6 months survival. Liver cirrhosis classified by Child-Pugh Class (points), MELD score, and CLIF-C-ACLF score. PSE = partial splenic embolization, MPN = myeloproliferative neoplasm, pEVH = persistent esophageal variceal hemorrhage, pGVH = persistent gastric variceal hemorrhage, rEVH= recurrent esophageal variceal hemorrhage, cEVH = controlled esophageal variceal hemorrhage with high risk of recurrent bleeding, cGVH = controlled gastric variceal hemorrhage with high risk of rebleeding, PHG = recurrent portal hypertensive gastropathy bleeding, NSBBs = nonspecific beta-blockers, PES = postembolization syndrome
